# Menopause-related changes to maxillary trabecular bone micro-architecture

**DOI:** 10.3389/fragi.2025.1589708

**Published:** 2025-05-08

**Authors:** Alexandra Stein, Michael Levit, Hammaad Shah, Michael Yin, Sunil Wadhwa

**Affiliations:** ^1^ College of Dental Medicine, Columbia University Irving Medical Center, New York City, NY, United States; ^2^ Vagelos College of Physicians and Surgeons, Columbia University Irving Medical Center, New York City, NY, United States

**Keywords:** orthodontics, maxilla, bone density, menopause, cone beam computed tomography

## Abstract

The number of midlife women seeking orthodontic treatment has significantly increased over the past 40 years. With this rise, orthodontists need to consider the potential impact of menopause on treatment planning. There have been no recent published studies on maxillary trabecular bone changes in humans related to menopause. This study aimed to explore the subject further. This cross-sectional cohort study was composed of qualifying participants with diagnostic maxillary CBCT images who were separated by self report into pre- (N = 21) and postmenopausal (N = 19) groups. The regions of interest were the trabecular bone of the incisive foramen and maxillary tuberosity. All scans were converted into binary images in order to draw all parametric and ratio raw data. The parameters of interest included trabecular bone volume fraction (BVF), trabecular thickness, trabecular number, and trabecular separation. In the incisive foramen subgroup, postmenopausal women showed a significant increase in trabecular separation (0.60 ± 0.25 to 0.84 ± 0.31 mm, P < 0.06). For the maxillary tuberosity subgroup, significant decreases in BV/TV (32.58 ± 15.85 to 17.63 ± 14.38 %, P <0.004), trabecular bone surface/tissue volume (2.66 ± 1.01 to 1.43 ± 1.09 %, P < 0.001) and trabecular separation (0.91 ± 0.39 to 1.58 ± 0.51 mm, P < 0.001) were observed. The findings reveal statistically significant differences in maxillary bone density at the level of the maxillary tuberosity and incisive foramen demonstrated in women who are of preversus post-menopausal status.

## Introduction

Our profession has had a significant increase in the number of adult orthodontic patients in the past 40 years (Khan and Horrocks, 1991). Between 2016 and 2018, at least 60,000 adults in the United States began orthodontic treatment (Hung et al., 2023). With a rise in the number of midlife women seeking treatment, the orthodontist must consider how menopause might impact treatment planning.

Menopause is defined as a transitional period, beginning 1–2 years prior to the last menstrual period (Gatenby and Simpson, 2024). This transitory phase is known as “perimenopause.” One of the largest meta-analyses on the topic shows that across the world the overall mean menopausal age was 48.8 years, ranging from 46–52 years (El Khoudary, 2020). Not all women assigned female at birth will experience symptoms, but up to 25% report physical and psychological symptoms including but not limited to low mood, altered sleep patterns, hot flushes, night sweats, and joint aches. As the average life expectancy for women is increasing, women may be menopausal for up to a third of their life (Gatenby and Simpson, 2024).

Menopause is the result of a depletion of ovarian follicles due to hormonal changes throughout a duration of years. It starts with a decline in levels of inhibin B, leading to a decrease in the negative feedback loop of follicle-stimulating hormone (FSH) release from the pituitary. As a result of this, FSH increases, which increases estrogen secretion. Over time, the ovary becomes less responsive to FSH during the menopausal period, which leads to reduced estrogen production. This results in a loss of luteinizing hormone stimulation, resulting in the inability to ovulate. The change in hormonal environment culminates into the series of symptoms seen within a female’s peripheral and central nervous system for an unpredictable amount of time (Monteleone et al., 2018).

Research has shown that estrogen is likely the major systemic regulator of bone metabolism not only in women, but also in men, suggesting that estrogen plays a universal role in bone metabolism (Khosla et al., 2012). Treatment of postmenopausal women with estrogen has been shown to lead to a reduction of serum and urine markers of bone resorption. A study by Lindsay and colleagues demonstrated that treatment of oophorectomized women with synthetic estrogen mestranol completely prevented decreases in metacarpal mineral content over 5 years *versus* the placebo-treated group, which showed a significant decrease (Lindsay et al., 1976). Bone biopsy data, obtained typically months to years after beginning estrogen replacement, has also shown a decrease in indications of bone resorption (osteoclast numbers) and formation rates (osteoblast numbers) (Khosla et al., 2012).

The current literature about the molecular mechanisms of action of estrogen deficiency show that estrogen binds with the estrogen receptor to promote expression of osteoprotegerin, and to suppress the action of nuclear factor-*κ*β ligand (RANKL). This inhibits osteoclast formation and bone resorptive activity. Estrogen can also activate the Wnt/β-catenin pathway to increase osteogenesis, promoting mesenchymal stem cell differentiation from pre-osteoblasts to osteoblasts (Cheng et al., 2022). Therefore, the loss of estrogen during menopause significantly increases the rate of bone resorption leading to a decrease in bone mass, resulting in post-menopausal osteoporosis, the most common type of osteoporosis (Eastell et al., 2016).

The effects of estrogen depletion are not limited to the perimenopausal period. The rate of bone mineral density loss in the long bones increases two to 3 years before the final menstrual period, and slows three to 4 years after (El Khoudary et al., 2019). The effects of estrogen depletion have not been as well studied on the craniofacial bones, in contrast to the abundance of research that exists surrounding menopause’s influence on the long bones. During human development, the long bones are derived from mesoderm while the anterior craniofacial bones, such as the maxilla, are derived from neural crest cells (Omi and Mishina, 2021). Also in contrast to the long bones which form by endochondral ossification, the maxilla forms by intramembranous ossification of bilateral plates of bone. Prenatally, the primary driver of the growth of the maxilla is the nasal septum. After birth, most of the nasal septum ossifies and the maxillary midline synchondrosis converts to the palatal suture. The nasal septum and palatal suture are considered “primary growth centers,” which result in vertical displacement and increase in width of the maxilla. Once molar occlusion is fully established at about 12–18 months of age, secondary growth sites then drive growth by responding to functional loading throughout life. This change in growth from primary to secondary mechanism is a unique feature of the trabecular bone in the craniofacial complex.

Likely due to the fact that it contains more “reactive trabecular bone,” the maxilla has been found to be even more responsive than the mandible to orthopedic and dentoalveolar change (Roberts and Hartsfield, 2004). When focusing on the trabecular bone in the craniofacial region, studies have shown evidence of region specific effects on the mandibular bone in postmenopausal women. These effects are shown to be more significant at the mandibular condyle, and less so on the mandibular alveolar bone (Levit et al., 2023). Another study by Munakata et al. showed a decrease in quality and quantity of trabecular bone in the mandibular molar region of post-menopausal women (Munakata et al., 2011).

In contrast to the mandible, there have been no recent published studies showing maxillary trabecular bone changes in humans related to menopause. A study done by White and Rudolph from 1999 was able to display a morphologic pattern change in osteoporotic patients *versus* a control patient in the anterior maxilla using periapical radiographs. However, the study was done utilizing conventional dental radiographs from multiple dental offices, resulting in limitations in findings due to standardization in image taking and processing conditions (White and Rudolph, 1999). A few studies on the topic have been done utilizing animal models. Ejiri et al. discussed effects of estrogen deficiency in the trabecular jaw bones and alveolar bone in ovariectomized rats and monkeys, stating that “jaw bones are equally [as] vulnerable to osteoporosis” as the other skeletal bones (Ejiri et al., 2008). Another study showed that osseointegrated maxillary dental implants in ovariectomized rats had a decrease in bone density compared with a sham-surgery group (Giro et al., 2011). Ishihara et al.‘s study also was conducted on ovariectomized rats. Their study found more prominent bone loss in bones formed by endochondral ossification, as opposed to the maxilla which is formed by intramembranous ossification (Ishihara et al., 1999). These animal studies suggest that similar changes could occur in the human trabecular maxillary bone of postmenopausal patients as well.

Considering the substantial developmental differences between the craniofacial complex and the long bones, along with the limited knowledge about menopause’s effects on the human maxillary trabecular bone, our objective was to gather more information on this topic. We aimed to analyze CBCT images to study standardized sections of the trabecular bone of the maxilla in individuals who have been identified as pre- or post-menopausal. It was hypothesized that based on the well known effects of perimenopause on long bones, that in the maxilla we should also find a decreased density of trabecular bone after the menopausal period. This could have a multitude of implications on our orthodontic treatment. If the maxillary bone density changes after the menopausal period, we could anticipate effects to the rate of tooth movement, our anchorage considerations, and bone loss during treatment in a postmenopausal patient population.

## Materials and methods

The protocol for this cross-sectional cohort study was approved by the Columbia University Irving Medical Center Institutional Review Board (#AAAR5233). All participants in the study provided written informed consent.

The sample was composed of patients who were part of a larger cohort recruited from the general dental clinic at CUIMC through IRB approved flyers and the RecruitMe website. Enrollment was monitored to balance sex and race. Specifically, these were individuals who were HIV-, pre, peri, or postmenopausal, and had CBCT scans that included the maxilla. Inclusion criteria included 1) female sex and 2) age range ≥35 years and ≤70 years. The exclusion criteria were 1) current chemo- or immunotherapy, 2) history of osteoporosis therapy (including but not limited to use of bisphosphonates), 3) current pregnancy, 4) currently nursing, and 5) current use of hormonal birth control. Qualifying participants with diagnostic maxillary CBCT images were separated into pre- and postmenopausal groups. Participants were considered postmenopausal if the last self-reported menstrual period was at least 1 year prior and if the individual self-reported to be of postmenopausal status. Blood samples were collected using serum separator tubes, separated into serum aliquots, stored at −80°C, then thawed and batch-analyzed at the Columbia University Irving Medical Center Biomarker Laboratory. Circulating estrogen levels were measured by Estradiol ELISA (Siemens Cat# LKE21).

### CBCT images

High resolution cone beam computed tomography (CBCT) images of the maxillary alveolar bone were obtained by a Planmeca ProMax 3D Classic CBCT scanner (Planmeca Inc., Hoffman Estates, Illinois, United States) at 84 kVp, 8 mA, and 15 s scan time. The voxel size was 150 mm. Participants were positioned in the scanner and secured using a temporal bone support and chin rest to reduce motion artifacts, and instructed to occlude on the posterior dentition in the position that provided the best fit. The aim was to obtain maximum occlusion. The manufacturer’s standard high resolution scanning protocol was used to acquire a diameter (Ø) 8 × 8 cm of field of view (FOV) at a nominal isotropic resolution of 150 μm, with images generated in sagittal and axially-corrected coronal views.

**FIGURE 1:**
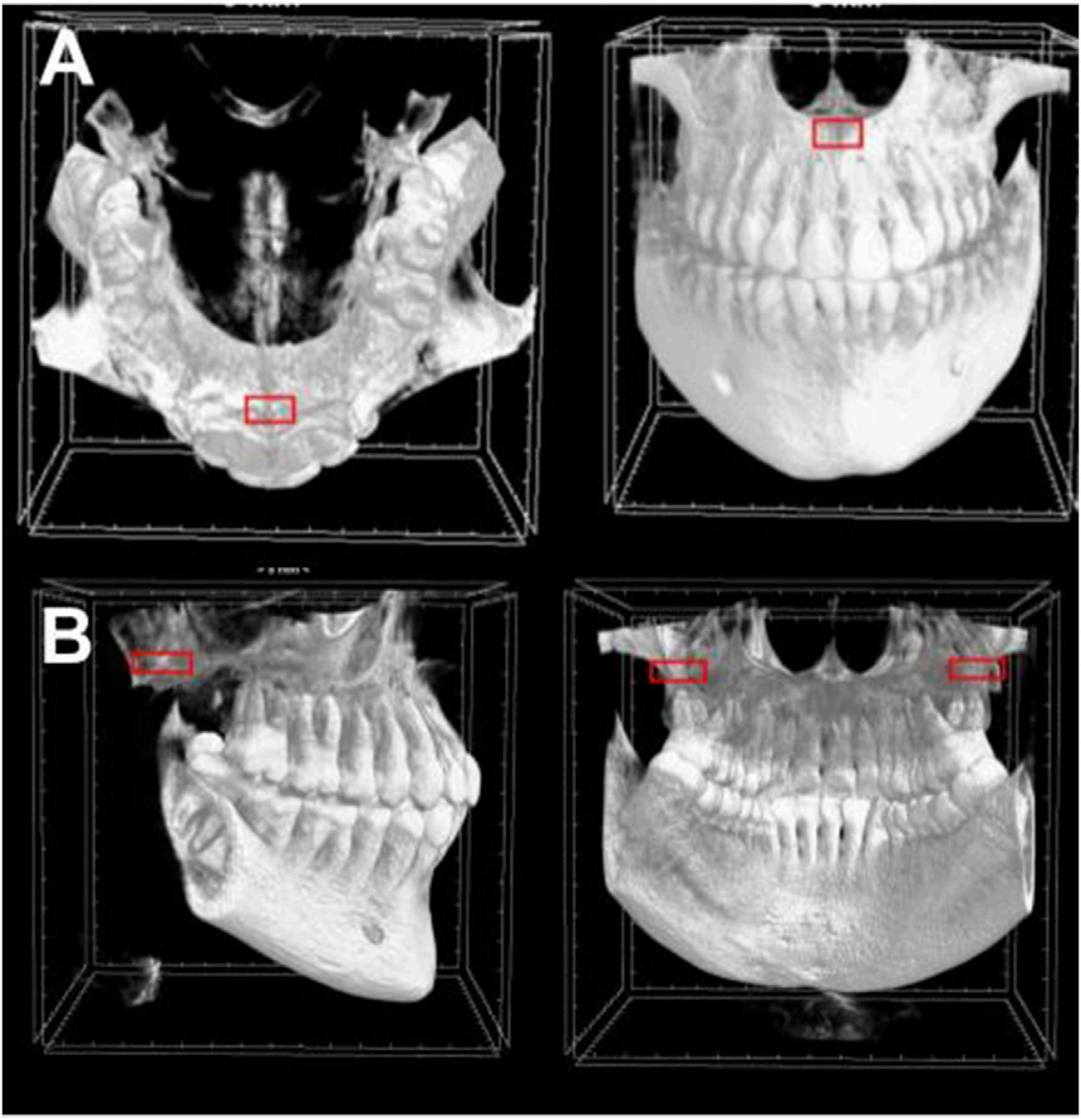
Regions of interest in CBCT sections. The region of interest box is demonstrated in red of **(A)** the incisive foramen and **(B)** the maxillary tuberosity.

The regions of interest were trabecular bone of the incisive foramen, and trabecular bone of the maxillary tuberosity. To analyze the maxillary tuberosity, fifteen consecutive sections without intersection gaps (2.25 mm) were stacked beginning at the most inferior portion of the maxillary sinus. A region of interest box measuring 15 × 7.5 mm was centered on the widest portion of the tuberosity for both sides and averaged. To analyze the incisive foramen, thirty consecutive sections without intersection gaps (4.5 mm) were stacked superiorly to where the incisive foramen becomes one concentric circle in the midline. A region of interest box measuring 4.5 × 2.25 mm was centered on the anterior of the foramen (Figure 1).

**FIGURE 2:**
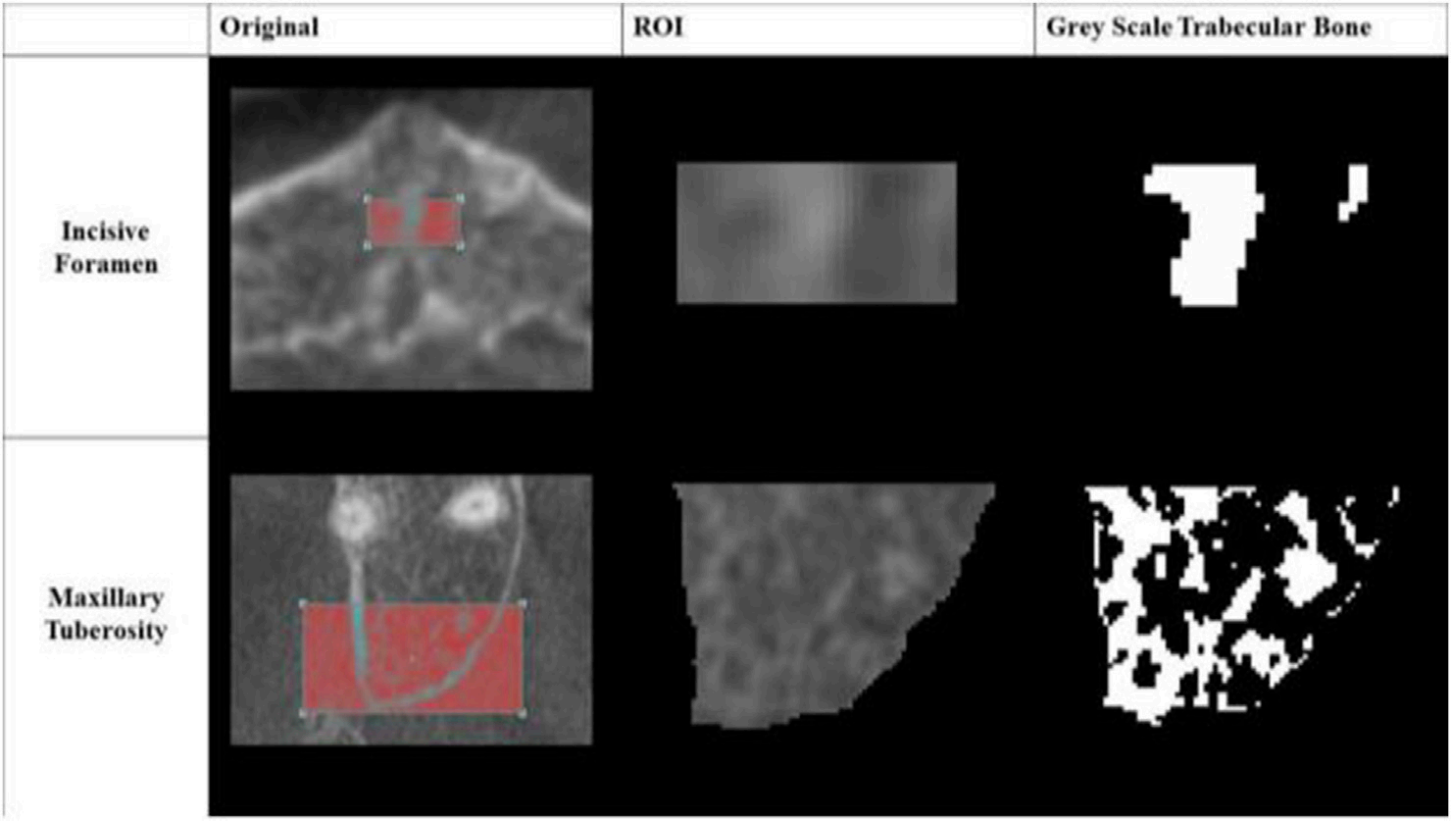
Greyscale Images of Regions of Interest (ROI). An example of each converted grayscale image is shown in its binary form for both the incisive foramen and maxillary tuberosity regions of interest.

The trabecular compartments were segmented with semi-automation from the CBCT scans in CT Analyzer (V1.15.4.0+, 2003–2011 SkyScan, Bruker). A specimen-specific automatic local threshold was then applied to convert the grayscale images into binary images (Figure 2). A fixed value of 39% of maximal gray-scale value was used for both maxillary tuberosity and incisive foramen regions of interest.

Parameters of interest included trabecular bone volume fraction (BVF), trabecular thickness, trabecular number, and trabecular separation. All parametric and ratio raw data were calculated by Skyscan CtAN. Axial images were isolated from the DICOM file using Skyscan Dataviewer.

### Statistical analysis

Our primary outcome was trabecular bone volume fraction (BVF). From our preliminary data, we found that mean and standard deviation of maxillary incisive trabecular BVF was 72.05% ± 18.9%. Therefore, for a sample size of 40, we calculated with a power of 80% that the study will be able to detect >25% differences between premenopausal *versus* postmenopausal groups at a two-sided 0.05 significance level.

All statistical analyses were conducted with Microsoft Excel (Microsoft Corporation 2013). Between-group differences in continuous measures were assessed with unpaired t-test and ANOVA, and demographic variables were assessed by Chi-square. Data are presented as means ± standard errors or n (%). Statistical significance was recorded at P < 0.05.

## Results

Sixty-two participants met the study’s inclusion and exclusion criteria. 31 were identified as being premenopausal, and 31 were identified as being postmenopausal (Table 1). Of the premenopausal group, 23 had available maxillary scans. For the postmenopausal group, 21 had available maxillary scans. Once excluding scans with artifacts in the regions of interest, the study resulted 21 premenopausal and 19 postmenopausal participants in the incisive foramen subgroup, and 20 premenopausal and 19 postmenopausal participants in the maxillary tuberosity subgroup.

**TABLE 1:** Cohort demographics for the incisive foramen and maxillary tuberosity subgroups.

Full cohort					
Variable	# Missing	All	Premenopausal	Postmenopausal	p-value
N		40	21	19	
Age	0	51.1 (10.2)	44.7 (7.5)	58.3 (7.8)	5.24E-05
Race/Ethnicity	0				0.992
African American		10 (25%)	5 (23.8%)	5 (26.3%)	
Hispanic White		19 (47.5%)	9 (42.9%)	10 (52.6%)	
Hispanic African		3 (7.5%)	2 (9.5%)	1 (5.26%)	
White		8 (20%)	5 (23.8%)	3 (15.8%)	
Smoker	0	6 (15%)	2 (9.5%)	4 (21.1%)	0.595
Diabetic	1	1 (2.56%)	0 (0%)	1 (7.1%)	N/A
Estradiol (pg/mL)	3	37	87.40 (76.01)	41.81 (57.41)	0.044

Cohort Variables are presented as mean ± SD or n (%). Missing values are indicated in the corresponding column for subjects lacking demographic data for a given variable. Smoking status represents current smoker when participants are enrolled. P < 0.05.

In the incisive foramen subgroup, a statistically significant increase (P < 0.01) was demonstrated in trabecular separation, from 0.60 ± 0.25 mm in the premenopausal group to 0.84 ± 0.31 mm in the postmenopausal group. A significant decrease (P < 0.003) in trabecular number was also found, from 0.77 ± 0.15 1/mm in the premenopausal group to 0.60 ± 0.18 1/mm in the postmenopausal group. Trabecular bone volume/tissue volume (BV/TV) (%) decreased appreciably from the pre-to postmenopausal group, but was not statistically significant (P < 0.06) (Table 2).

**TABLE 2:** Parametric results for the incisive foramen and maxillary tuberosity subgroups.

Variable	# Missing	Premenopausal	Postmenopausal	p-value
Bone Parameters Incisive Foramen		N = 21	N = 19	
Trab BV/TV (%)		72.05 (18.90)	59.74 (20.89)	0.06
Trab Thickness (mm)		0.95 (0.25)	0.95 (0.18)	0.96
Trab Separation (mm)		0.60 (0.25)	0.84 (0.31)	**0.01**
Trab Number (1/mm)		0.77 (0.15)	0.60 (0.18)	**0.003**
Bone Parameters Maxillary Tuberosity	1	N = 20	N = 19	
Trabecular BV/TV (%)		32.58 (15.85)	17.63 (14.38)	**0.004**
Trabecular Thickness (mm)		0.53 (0.15)	0.51 (0.15)	0.711
Trabecular Separation (mm)		0.91 (0.39)	1.58 (0.51)	**<0.001**
Trabecular Number (1/mm)		0.60 (0.24)	0.33 (0.26)	**0.002**

Missing values are noted in the corresponding columns for scans with artifacts in the region of interest. Bone parameters among data are presented as mean ± SD or n (%). P < 0.05.

For the maxillary tuberosity subgroup, a statistically significant decrease (P < 0.004) was demonstrated in the trabecular BV/TV, from 32.58% ± 15.85% to 17.63% ± 14.38%. Trabecular separation had a significant decrease from pre-to postmenopausal (P < 0.001) as well, from 0.91 ± 0.39 mm to 1.58 ± 0.51 mm. Significant decline (P < 0.002) was similarly found in the trabecular number, from 0.60 ± 0.24 1/mm to 0.33 ± 0.26 1/mm. No significant differences were demonstrated in trabecular thickness (mm) (Table 2).

## Discussion

Understanding how the bone architecture alters with age is crucial in the current age of orthodontics, especially as the average age of our patient population continues to increase. In this study, we examined the bony changes in the maxilla of premenopausal *versus* postmenopausal women, focusing on two specific regions: the posterior maxilla at the maxillary tuberosity and the anterior maxilla at the incisive foramen utilizing measurements made from CBCT imaging. These reference points were selected due to their reliability as standardized and reproducible regions of interest, as they are landmarks common to the entire patient sample.

The current clinical diagnosis for menopause in healthy women over 45 years old is to have not had a period for at least 12 months and are not using hormonal contraception, or who do not have a uterus and have menopausal symptoms. Women in our study were considered postmenopausal if they reported post-menopause status and the date of their last menstrual period was more than 1 year ago. This definition of menopause is consistent with the recommendations on the diagnosis (Colditz et al., 1987). Estradiol levels, sampled from all participants in the study, were found to have a significance level of P < 0.05 between groups, further supporting the pre-vs. post-menopausal group classification.

At the anterior region of the maxilla at the level of the incisive foramen, our study showed a significant decrease (P < 0.01) in the trabecular separation from pre-to post-menopausal groups. This correlated with the significant decrease our study also found between groups for trabecular number (P < 0.003) and appreciable, but not statistically significant differences (P < 0.06) for BV/TV. There is no existing literature on trabecular maxillary bone in a postmenopausal group for comparison. However, these findings are similar to a study done by Naghibi et al., who focused on the changes in cortical buccal bone thickness measurements in the aesthetic zone (maxillary canine to canine) of pre vs. postmenopausal women through CBCT analysis. In their cross-sectional study, they found a statistically significant difference in buccal bone thickness at the right canine between groups. The mean anterior maxilla buccal bone thickness was higher in premenopausal women, but not statistically significant (Naghibi et al., 2022).

At the posterior maxilla, identified by the maxillary tuberosity, our study found significant decreases in BV/TV (p < 0.004), trabecular number (p < 0.002) and separation (p < 0.001). The significant difference we found in both the anterior and posterior maxilla are related to findings by Ko et al., who only found a significant difference in crestal cortical bone in the posterior maxilla vs. anterior maxilla with age (Ko et al., 2020). Though our study found significant decreases in bone density in both the posterior and anterior maxilla, more statistically significant decreases were found at the level of the maxillary tuberosity in the posterior maxilla.

The finding that maxillary bone quality does have a significant decrease in trabecular bone density in a woman after menopause is of high clinical relevance. A recent meta-analysis reviewing studies which included CBCT data demonstrated that in the anterior maxilla, bone loss can be expected of up to 0.94 mm (Guo et al., 2021). Another study, also using CBCT analysis, found decreased bone densities of up to 24% after just 7 months of orthodontic treatment (Hsu et al., 2011). If bone loss is already a result of orthodontic treatment on non-menopausal patients, the fact that it might be increased due to the results of expected estrogen depletion in a female may warrant us as orthodontists to reconsider our treatment mechanics in a female patient population of postmenopausal status. Studies show that bone density is inversely related to rate of tooth movement (Chugh et al., 2013). Therefore, if bone density reduces due to menopause, one could assume that the rate of tooth movement in a postmenopausal female would increase. In addition, if post-menopausal status enhances the bone loss that already naturally occurs due to orthodontic tooth movement, an orthodontic provider may want to consider a less invasive or more time efficient approach to treatment in order to avoid additional bone loss to the periodontium. An orthodontist may want to avoid certain orthodontic movements such as upper incisor retraction. This is because if the maxillary bone density is reduced, the postmenopausal population may be more susceptible to bony fenestrations due to buccally torqued maxillary incisor roots (a side effect of incisor retraction).

A large limitation to our study was the small sample size. Considering the number of scans available for analysis and the resultant number after excluding artifacts, it could be possible that the findings which were appreciable but not statistically significant such as the values for BV/TV in the anterior maxilla might trend toward statistically significant with a larger sample size. This challenge has also been observed in other studies on the topic, where findings were of appreciable statistical value but not significant, likely due to only a small sample size being available (Naghibi et al., 2022). In addition, the fact that our study was cross-sectional as opposed to retrospective or prospective, eliminated our ability to control confounding factors between individuals which may affect bone quality over time. Confounding variables that could be of note are race, ethnicity, and socioeconomic status (Schenkein et al., 1993). A prospective study would allow us the ability to observe a temporal sequence of events and better assess causality. Finally, it is difficult to confirm that all subjects who identified as pre-menopausal were not already in the perimenopausal stage, as there is currently no precise method to determine whether a woman is in the transitional 1–2 years preceding her final menstrual period with our menopause definitions (Bastian et al., 2003).

Acknowledging these limitations in our study, future research on this topic should ideally include a larger sample size, with more CBCTs available for analysis. These studies could be conducted prospectively, with CBCT focus on specific regions of interests at the maxillary tuberosity and incisive foramen over time, as female patients transition from pre to postmenopausal status.

## Conclusion

- A statistically significant difference in maxillary bone density at the level of both the maxillary tuberosity and incisive foramen is demonstrated in women who are of pre *versus* postmenopausal status
- Prospective research with larger sample sizes would add value to the findings of this study

## Data Availability

The original contributions presented in the study are included in the article/supplementary material, further inquiries can be directed to the corresponding author.
